# Can an Unenhanced Reduced-Dose ECG-Gated CT of the Aorta Replace an ECG-Gated CT-Angiography for Diameter Follow-Up of the Ascending Aorta?

**DOI:** 10.3390/jcdd13050176

**Published:** 2026-04-24

**Authors:** Thomas Saliba, Denis Tack, Nicolas Naccarella, Sanjiva Pather, David Rotzinger, Olivier Cappeliez

**Affiliations:** 1 Radiology Department, Centre Hospitalier Universitaire Vaudois (CHUV), 1011 Lausanne, Switzerland; david.rotzinger@chuv.ch; 2 Radiology Department, Centre Hospitalier Interrégional Edith Cavell (CHIREC), 1420 Braine-L’Alleud, Belgium; nicolas.naccarella@chirec.be (N.N.); sanjiva.pather@chirec.be (S.P.);; 3 Radiology Department, Hopital Erasme, 1070 Brussels, Belgium; 4 Radiology Department, Hopital Epicura, 7800 Ath, Belgium

**Keywords:** CT, radiation protection, thoracic aortic aneurysm, CTA

## Abstract

Electrocardiogram (ECG)-gated contrast-enhanced computed tomography angiography (CTA) is the reference method for follow-up of ascending aortic aneurysms but delivers substantially higher radiation doses than ECG-gated non-contrast CT (NCCT). NCCT can be acquired at a lower dose while enabling measurements of the aortic outer diameter. This study aimed to quantify the radiation dose of both techniques and determine whether a significant difference exists in ascending thoracic aorta diameter measurements between NCCT and CTA. Eighty patients who underwent ECG-gated cardiac CT for suspected coronary artery disease were retrospectively analyzed. Three observers measured the ascending aortic diameter at the level of the pulmonary artery in a plane perpendicular to the aorta on both NCCT and CTA images. Inter-rater reliability was assessed using intraclass correlation coefficients, and paired samples t-tests were used to evaluate measurement differences. Dose-length products (DLP) were collected. Median DLP values were 16.1 mGy·cm (interquartile range 11.8–25.1) for NCCT and 190.3 mGy·cm (interquartile range 120.5–298.9) for CTA. NCCT measurements were consistently larger than CTA measurements, with mean differences of 2.1 ± 0.8 mm, 2.6 ± 0.96 mm, and 2.9 ± 1.09 mm for the senior radiologist, junior radiologist, and resident, respectively (all *p* < 0.001). Inter-observer agreement was excellent (ICC = 0.99, *p* < 0.001). NCCT delivered an 11.8-fold lower radiation dose than CTA. NCCT may replace CTA for ascending aortic diameter follow-up if measurements are adjusted by approximately 2–3 mm relative to CTA-derived inner-diameter thresholds.

## 1. Introduction

Aortic aneurysms represent the most common thoracic aorta pathology, with an incidence of 3.6 to 7.6/100,000 [1]. Ascending thoracic aorta measurements are performed to detect aneurysms. Intervention is generally indicated at 55 mm, although some surgical groups and certain pathologies consider 50 mm at the aortic root as the threshold [2,3]. With these guidelines in place, accurate and reproducible aortic measurements are crucial. This is, however, a challenging endeavor; region-specific issues result in discrepancies due to variations in measurement techniques, all of which yield slightly different values [2,4,5]. ECG-gated CT acquisitions can mitigate issues linked to the heart cycle, though most aneurysms are incidental findings on non-contrast, non-ECG-gated CT scans [2,6,7].

While ECG-gating addresses pulsatility as a source of measurement error, the use of intravenous contrast is an equally important, though less discussed, source of variability that fundamentally alters the measurement. Contrast-enhanced CT is preferred for specific indications for aortic imaging because it allows delineation and assessment of the aortic lumen. However, non-contrast CT (NCCT) is a viable alternative for measuring aneurysm diameter when contrast is unavailable or unsuitable [8]. Typically, only the lumen will be measured on contrast-enhanced CT scans, because the wall may be poorly visualized [2]. On the other hand, NCCT will result in measurements including both the lumen and the wall, potentially leading to variations of up to 6 mm between the two techniques [2].

The choice of modality carries important implications for patient safety. Studies have shown that CTA examinations result in significantly more radiation dose than NCCT and calcium-score acquisitions [9,10]. A clinical study of chest CT scans found that patients receiving intravenous contrast exhibited twice as much DNA damage in peripheral blood lymphocytes as those who did not, highlighting a significantly higher potential cancer risk [11]. This is all the more relevant for populations with aortic aneurysms as they undergo regular follow-up examinations and are thus frequently subject to irradiating examinations [12]. There is therefore a pressing need to optimize thoracic aortic aneurysm follow-up examinations to prevent excessive radiation and subsequent harm. Because unenhanced ECG-gated CT (NCCT)—typically used for calcium scoring with prospective gating and sequential mode—delivers lower doses than ECG-gated CT angiography (CTA), frequently acquired in helical mode and retrospective gating, it would be preferable to use NCCT instead of CTA for repeated follow-up of aortic diameter.

Previous studies have reported possible differences in ascending thoracic aorta diameter between ECG-gated NCCT and ECG-gated CTA, though none have specifically studied and quantified the difference as their primary aim [12]. Consequently, the primary aim of this retrospective study is to determine whether a significant difference exists in the diameter of the ascending thoracic aorta when measured on ECG-gated NCCT versus ECG-gated CTA, and to quantify the average magnitude of this difference. The secondary aim is to quantify the radiation doses delivered by NCCT and CTA.

## 2. Materials and Methods

### 2.1. Patient Population

This retrospective single-center study included 80 consecutive patients with no history of thoracic aortic surgery who underwent ECG-gated cardiac CT for suspected coronary artery disease. All CT examinations were identified in the picture archiving and communication system (PACS). The patient population consisted of 57 men and 23 women. The average age of the patients was 60 ± 9.9 years old. We did not control for atheroma, calcification or wall thickening, though none of the patients in our series suffered from this. We did neither control for arrhythmias nor atrial fibrillation; however, none of the patients in our series were known to have these conditions. All of the acquisitions of the 80 consecutive patients were of good quality.

### 2.2. Computed Tomography Protocol

All CT examinations were performed on a Somatom Definition Edge (SIEMENS Healthineers, Erlangen, Germany) system. The prospectively ECG-gated NCCT calcium scoring acquisition was performed using sequential acquisition at 120 kV and variable current, with reconstructed 3 mm thickness slices available on the PACS. The retrospectively ECG-gated CTA was performed following the intravenous bolus injection of iodinated contrast medium (IOMERON 400 mg I/mL) flushed with saline, using a spiral acquisition with variable kV and mA that were automatically defined by the device (CareDose 4D, Siemens, Munich, Germany), with retrospectively reconstructed 3 mm thickness slices available on the PACS. Both NCCT and CTA scans were reconstructed in the same phase to eliminate aortic pulsatility as a confounding factor.

### 2.3. Observers

Three researchers, a senior cardiac radiologist (>15 years of experience), a junior cardiac radiologist (>5 years of experience), and a resident (>200 cardiac-CT examinations performed under supervision), retrospectively examined all 80 ECG-gated cardiac-CT examinations, including non-contrast and contrast-enhanced axial ECG-gated images of the ascending thoracic aorta.

### 2.4. Measurement Software

The CT scans were assessed, and the measurements were performed using Enterprise Imaging software version 8.8.2 050 (AGFA Healthcare NV, Mortsel, Belgium).

### 2.5. Measurements

**Figure 1:**
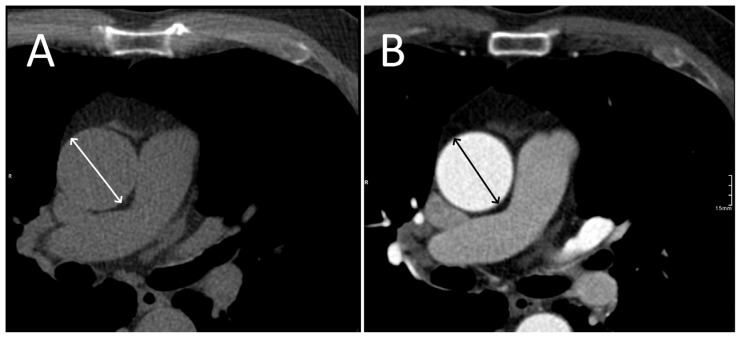
Axial NCCT (**A**) and CTA (**B**) images demonstrate the image alignment used for the measurements. This figure illustrates a 2 mm disparity between the NCCT and CTA measurements. The arrows represent the diameter of the aorta.

Each researcher measured the largest measurable diameter of the ascending aorta in the axial plane, at the level of the common pulmonary artery, on both the CTA and NCCT images. This plane was chosen because it produces a section perpendicular to the aorta and is generally considered sufficient for screening purposes [13]. Prior to measurement, the datasets were co-registered to ensure alignment, and the same aortic axis was used by agreement. Using multiplanar reconstructions from the CTA, we verified that the selected plane was perpendicular to the aorta and not oblique by comparison with coronal and sagittal views. Three-millimeter thickness reconstructions were used to maintain uniformity between studies and NCCT and CTA exams. The systolic or diastolic phase of the heart was matched between acquisitions to eliminate phase as a confounding factor (Figure 1). The exams used were cardiac CTs in order to have both ECG-gated NCCT and CTA acquisitions and ensure the greatest degree of comparability between NCCT and CTA acquisitions.

To eliminate the reconstruction kernel as a confounding factor, we conducted a series of tests in which NCCT and CTA images were reconstructed using different kernels. This exposed no differences in the measurements between the NCCT measurements and CTA measurements with different reconstructions, showing that the difference between the two acquisition types remained despite varying the kernels, enabling us to eliminate a potential kernel-related bias.

An aortic diameter ≥ 40 mm was defined as aortic dilatation, and measurements ≥ 45 mm were considered aneurysms [8].

### 2.6. Radiation Dose

The dose-length products of all acquisitions were collected using a DACS (data archiving and communicating system) (Intuitus, Telemis, Ottignies-Louvain-la-Neuve, Belgium).

### 2.7. Statistical Analysis

The results were analyzed using IBM SPSS Statistics version 28.0.1.1 (14) (IBM, New York, NY, USA).

An intraclass correlation coefficient was used to determine the inter-rater reliability of the measurements between the researchers. In addition, a Bland–Altman analysis was performed to assess the agreement between both measurements.

Paired samples *t*-tests were used to determine differences in aortic diameter measurements between NCCT and CTA. A *p*-value of <0.05 was considered significant for all the statistical tests performed.

This study was approved by the institutional ethics committee.

## 3. Results

In total, 80 patients (corresponding to 80 ECG-gated NCCT and 80 ECG-gated CTA) were included in the study. Of those 80 patients, 30 (37.5%) were female. The average age was 63 years (±9.36) for females and 59.3 years (±10.00) for males.

The three observers performed a single measurement for each NCCT and CTA exam, with a total of 480 measurements. The mean difference between CTA versus NCCT measurements was 2.1 (±0.8) mm (*p* < 0.001), 2.6 (±0.96) mm (*p* < 0.001), and 2.9 (±1.09) mm (*p* < 0.001) for the senior radiologist, the junior radiologist, and the resident, respectively. The average measurements for the different observers are found in Table 1.

The inter-observer correlation was excellent, with values of 0.99 (*p* < 0.001) for single measures and 0.997 (*p* < 0.001) on average. A Bland–Altman analysis revealed a mean difference of 2.1 (±0.81), 2.57 (±0.96) and 2.88 (±1.09) for the senior, junior and resident radiologist respectively, showing no evidence of proportional bias.

Median DLP of NCCT was 16.1 (interquartile range: 11.8 to 25.1) mGy.cm and that of CTA was 190.3 (interquartile range: 120.5 to 298.9) mGy.cm, resulting in a radiation dose ratio of 11.8 (CTA vs. NCCT).

## 4. Discussion

We found that NCCT measurements of the ascending aortic diameter are consistently 2–3 mm larger than CTA measurements. This reflects the inclusion of the aortic wall on NCCT and its frequent exclusion on CTA due to limited wall definition and blooming from the contrast-filled lumen. We observed a 2.4 mm average difference (*p* < 0.001) in ascending aorta diameter between NCCT that included the wall and CTA exams without the wall [12]. We measured a mean difference ranging from 2.1 to 2.9 mm in aortic diameter between CTA and NCCT acquisitions, depending on the researcher. We also observed very low inter- and intra-observer variability in our aortic diameter measurements, showing that our methods are replicable.

**Figure 2:**
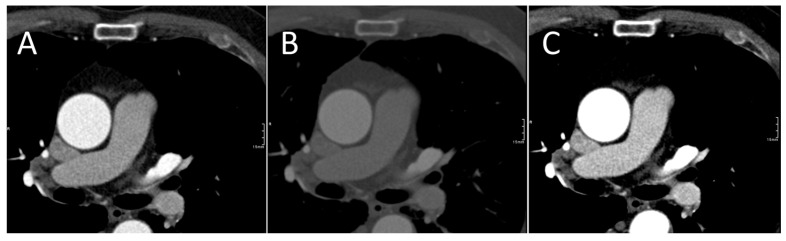
Axial images from an arterial CTA demonstrate the difficulty of clearly delineating the aortic wall in standard CTA windowing (W:1500, L:450) (**A**), bone windowing (W:2000, L:500) (**B**) and soft-tissue windowing (W:350, L:50) (**C**).

**Figure 3:**
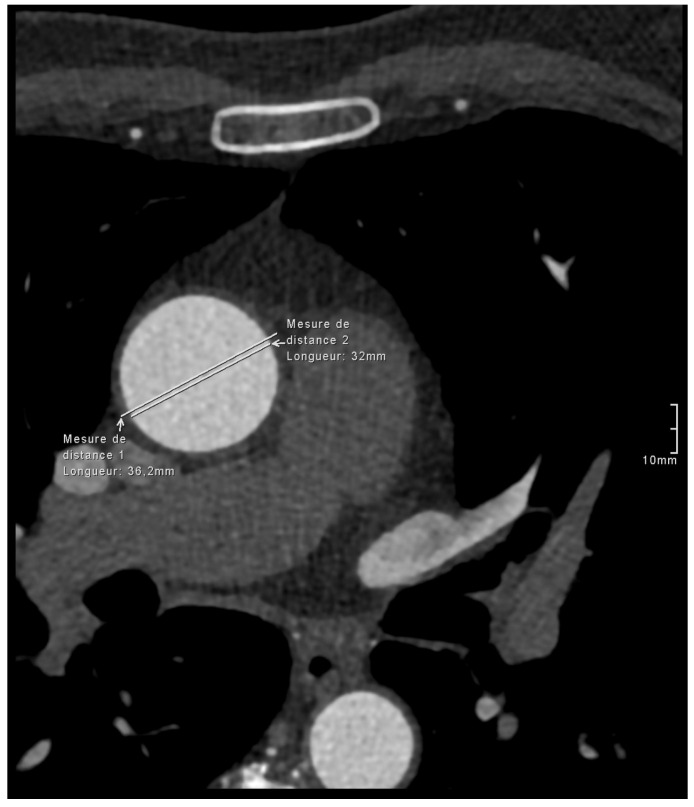
Axial image from a CTA exam of a patient with a thickened aortic wall, thus allowing aortic wall visualization, demonstrating a 4 mm discrepancy between the measurements, including and excluding the aortic wall.

One previous study reported similar findings, though they found no difference between the diameter measurements with the CTA, where they attempted to include the aortic wall and NCCT [12]. However, it is unclear how they were able to accurately measure the aortic wall on the CTA, as no figures were provided to explain their method. In practice, measurements on CTA examinations will exclude the aortic wall, whereas the wall is always included on NCCT measurements (Figure 2). Furthermore, obtaining measurements including the wall on CTA can be challenging due to variable image quality, making the wall visible on some acquisitions and not on others, regardless of the windowing (Figure 3).

Our results add another layer of complexity to the thoracic aorta measurement guidelines, which already vary significantly among different professional societies. The ACC/AHA (2022) and ESC (2024) guidelines recommend measuring the inner diameter unless aortic wall disease is present [3,14]. In contrast, the ACTS and STS (2024) guidelines advocate for outer-to-outer measurement, reflecting a clear discrepancy between surgical and non-surgical specialties [8]. Notably, the ESC guidelines also state that NCCT can substitute CTA when contrast is contraindicated, without acknowledging the potential for significant measurement discrepancies between the two modalities. Furthermore, it has been acknowledged that NCCT is sufficient to monitor ascending aortic diameter [15]. It should also be noted that the most current treatment guidelines are based on research using outer-wall to outer-wall measurements [16,17].

A significant advantage is that NCCT acquisitions have been shown to result in around 30% less ionizing radiation than CTA acquisitions [9]. In our study, this ratio was 11,8 because the NCCT acquisition mode was that of a calcium score protocol. This has been further shown by studies comparing effective doses received by patients undergoing CTA and ECG-gated prospective NCCT for calcium scoring, showing that NCCT reduced radiation exposure by factors of 4.8, 7.9 and 4.29 for examinations acquired at 100 kV, 120 kV and 140 kV, respectively [10]. It would therefore be feasible to acquire the entire thoracic aorta using a calcium score protocol at a fraction of the dose used for thoracic aorta CTA [18]. Various groups have demonstrated the viability of this technique, with studies recording average doses as low as 1.23 mSv for the entire thoracic aorta [18,19]. Using a conversion factor of 0.014 mSv/mGy.cm, our corresponding effective dose for NCCT would be 0.22 mSv, typically that reported for chest radiographic examinations [20,21]. Therefore, if we are to act in accordance with the ALARA (as low as reasonably achievable) principle, we should strive to achieve the required result whilst exposing the patient to the least radiation possible [22]. This difference in radiation dose has significant implications, given that patients will undergo regular scans due to the need to monitor aortic diameters, with guidelines recommending follow-ups every 6 to 36 months [23]. This is all the more important for patients with connective tissue disorders such as Marfan’s syndrome, who will begin follow-ups at a young age and will therefore have a high cumulative radiation exposure [24]. This has ethical implications in that avoiding needless radiation is in line with the key values of beneficence/non-maleficence and prudence, which all medical practitioners should strive to uphold [25].

Attempts have been made to reduce CTA dose by using both high-pitch and prospective gating. High-pitch acquisitions, where gantry rotation pitch is increased, have been facilitated by the development of dual-source CT scanners [26]. Studies using this technology have shown that DLP can be reduced by around 28% without degrading quality compared to standard dual-source acquisitions [26]. Other studies have looked at the ability of prospective gating to reduce radiation dose, proving that the use of this technology significantly lowers the patient’s exposure during CTA for both single and dual-tube CT scanners [21,27]. One recent study where low-dose CTAs were performed found that using a 100 kV acquisition and a tin filter met the dose-reduction goal [28]. Yet another recent study utilized high-pitch prospective ECG-gated acquisitions and a 70 kv or 100 Kv acquisition [29].

Another benefit of NCCT over CTA is the absence of intravenous contrast, eliminating the risk of contrast media extravasation, contrast-induced encephalopathy and acute kidney injury [30]. Arguably, the most severe complication is contrast-induced anaphylaxis, which was found to occur in 1.6 per 1000 patients according to one study [31]. This is all the more important due to the risk of biphasic reactions, which can be more severe and occur at up to 72 h after the initial reaction, thus requiring patient hospitalization [32]. Eliminating this risk is non-negligible, especially if it is unnecessary.

The public health costs associated with contrast-enhanced CT examinations must also be considered. The financial impact of CTA examinations is compelling when compared to NCCT examinations, with one study showing that thoracic CTs performed with contrast in the setting of lung cancer follow-ups cost on average $367 more [33]. Another recent study of common imaging examinations from the USA using data from 2023 found that the average NCCT cost $868, whereas CTA costs $1288 [34]. This is significant as these patients can undergo up to two scans a year, which may increase the annual screening costs by nearly $1000, and can be even more significant over the long term. This is particularly relevant in the context of rising costs of living putting pressure on patients to avoid healthcare due to financial reasons, with a recent survey from Belgium showing that half of the residents of the capital city of Brussels avoided care in the past year due to costs [35]. This trend exists on a larger scale, with 8.3% of residents avoiding healthcare due to costs in 2018, according to the CDC [36]. A more recent survey from the USA showed that a third of patients did not get healthcare due to costs, with the figure rising to 75% when focusing on the uninsured [37]. This, therefore, raises the question as to whether excessive use of CTA instead of NCCT is also negatively impacting patients on the grounds of healthcare equity, with the added moral complexity that the decision to add the extra cost lies with the radiologist.

Our study has several limitations. First, none of our patients had significant abnormal thickening or calcification in the measured segment of the tubular ascending aorta, so our findings may not apply to patients with pathological aortic walls. Furthermore, none of the patients suffered from arrhythmias, and thus the potential effects of such pathologies were also not assessed by our study. Second, our data were limited to scans from a single patient population, all acquired on the same CT system. Third, although we matched the acquisition phases, we did not attempt to assess whether a measurement difference existed in systole versus diastole. Fourth, though it was sufficient to show a statistically significant difference of *p* < 0.001, the sample size was relatively small at only 80 individuals. Fifth, the two acquisitions were different, with the CTA being retrospective and the calcium-score NCCT being prospective. Still, in a clinical context, factors such as irregular or elevated heart rates can prevent patients from undergoing prospective CTA; however, these conditions do not affect NCCT examinations or aortic diameter measurements. Sixth, only a single measurement was made by each observer for every exam, making us unable to calculate any intraobserver variabilities. Finally, we used a single-source CT device that does not enable high-pitch acquisitions as provided by dual-source CT devices. The delivered radiation dose of a high-pitch non-ECG-gated acquisition is known to provide accurate measurements of the aorta. Therefore, the 11,8-fold difference in DLP observed with our single-source CT would not be that observed with a dual-source CT and is likely to be device-dependent.

## 5. Conclusions

In conclusion, the systematic difference of 2–3 mm between ECG-gated NCCT and ECG-gated CTA does not justify the systematic use of CTA for follow-ups of aortic diameter. In some circumstances, the use of NCCT can replace CTA, resulting in an 11.8-fold dose reduction as well as significant economic savings linked to abstaining from the use of contrast.

## Figures and Tables

**Table 1:** Paired sample *t*-test showing the average largest diameter, standard deviation and range measurements by the three observers for both the CTA and NCCT exams.

Researcher	CTA	NCCT
Senior radiologist	33.3 (±4.3) range 25–50	35.3 (±4.3) range 26.3–52
Junior radiologist	32.8 (±4.2) range 25–48.7	35.4 (±4.3) range 27.2–51.9
Resident	32.7 (±4.4) range 24.8–50	35.7 (±4.3) range 26.5–51.4

## Data Availability

Data is available upon reasonable request.

## Abbreviations

The following abbreviations are used in this manuscript:

ACC	American College of Cardiology
ACTS	American Association for Thoracic Surgery
AHA	American Heart Association
ALARA	As Low as Reasonably Achievable
CAD	Coronary Artery Disease
CDC	Center for Disease Control and Prevention
CT	Computed Tomography
CTA	Computed Tomography Angiography
DACS	Data Archiving and Communication System
DLP	Dose Length Product
ECG	Electrocardiogram
ESC	European Society of Cardiologyµ
ICC	Intraclass Correlation Coefficient
kV	Kilovoltage
mA	Milliampere
mGy·cm	Milligray-centimeter
mSv	Millisievert
NCCT	Non-Contrast Computed Tomography
PACS	Picture Archiving and Communication System
SPSS	Statistical Package for the Social Sciences
STS	Society of Thoracic Surgeons
